# Treatment with Luteolin Improves Lipopolysaccharide-Induced Periodontal Diseases in Rats

**DOI:** 10.3390/biomedicines8100442

**Published:** 2020-10-21

**Authors:** Giovanna Casili, Alessio Ardizzone, Marika Lanza, Enrico Gugliandolo, Marco Portelli, Angela Militi, Salvatore Cuzzocrea, Emanuela Esposito, Irene Paterniti

**Affiliations:** 1 Department of Chemical, Biological, Pharmaceutical and Environmental Sciences, University of Messina, Viale Ferdinando Stagno D’Alcontres, 31-98166 Messina, Italy; gcasili@unime.it (G.C.); aleardizzone@unime.it (A.A.); mlanza@unime.it (M.L.); egugliandolo@unime.it (E.G.); salvator@unime.it (S.C.); eesposito@unime.it (E.E.); 2 Department of Biomedical and Dental Science, Morphological and Functional Images, University of Messina, Via Consolare Valeria, 98125 Messina, Italy; mportelli@unime.it (M.P.); amiliti@unime.it (A.M.)

**Keywords:** dental diseases, periodontitis, luteolin, flavonoids, lipopolysaccharide, anti-inflammatory

## Abstract

Periodontitis is a dental disease that produces the progressive destruction of the bone surrounding the tooth. Especially, lipopolysaccharide (LPS) is involved in the deterioration of the alveolar bone, inducing the release of pro-inflammatory mediators, which cause periodontal tissue inflammation. Luteolin (Lut), a molecule of natural origin present in a large variety of fruits and vegetables, possess beneficial properties for human health. On this basis, we investigated the anti-inflammatory properties of Lut in a model of periodontitis induced by LPS in rats. Animal model predicted a single intragingival injection of LPS (10 μg/μL) derived from *Salmonella typhimurium*. Lut administration, was performed daily at different doses (10, 30, and 100 mg/kg, orally), starting from 1 h after the injection of LPS. After 14 days, the animals were sacrificed, and their gums were processed for biochemical analysis and histological examinations. Results showed that Lut (30 and 100 mg/kg) was equally able to reduce alveolar bone loss, tissue damage, and neutrophilic infiltration. Moreover, Lut treatment reduced the concentration of collagen fibers, mast cells degranulation, and NF-κB activation, as well as the presence of pro-inflammatory enzymes and cytokines. Therefore, Lut implementation could represent valid support in the pharmacological strategy for periodontitis, thus improving the well-being of the oral cavity.

## 1. Introduction

Periodontal disease can be defined as an infectious–inflammatory process that affects anatomical structures supporting the tooth: gums, periodontal ligament, cement, and alveolar bone [1,2]. Periodontitis is the leading cause of tooth loss in the adult population of industrialized countries; thus, it represents a serious health problem that affects a great portion of the world’s population (more than 50%). It is generally more frequent in adults and the elderly, but some forms can also affect children and adolescents [3]. Predisposing factors are incorrect nutrition [4], cigarette smoking [5], and certainly poor oral hygiene [6], as well as a possible hereditary component [7]. However, the process of altering periodontal structures is always the consequence of the concurrent action of immunological and microbial factors [2]. The oral cavity is colonized by more than 600 species of bacteria [8]. Some of them are beneficial to the health; however, when the balance in the microbial flora of the oral cavity is altered, this can establish conditions that favor the onset of infection [9]. Specifically, bacteria responsible for periodontitis hold lipopolysaccharides (LPS).

LPS is one of the most important molecules involved in the development of periapical inflammation and deterioration of the alveolar bone; the increase in its concentration causes the release of a variety of pro-inflammatory mediators, including prostaglandins and cytokines, which cause periodontal tissues inflammation through the activation of multiple pathways [10]. Inflammatory condition implicates the stimulation of fibroblasts, the increase of collagen breakdown, and the rise of osteoclast activity [11,12].

Given the severity of the disease, it is certainly important to act promptly with effective therapy. Currently, the most suitable drugs in the case of periodontitis are anti-inflammatory drugs of both steroid and non-steroidal origin (NSAIDs) [13], as well as antibiotics [14] and antibacterial mouthwashes containing chlorhexidine [15]; all of this should be combined with proper oral hygiene. 

In the most advanced forms of periodontitis, surgical techniques are also required.

In addition to conventional drugs, natural compounds can also be a valuable aid, providing additional support in the management of many inflammatory diseases. 

Luteolin (Lut; 3′,4′,5′,7′-tetrahydroxyflavone) is a polyphenolic compound that belongs to flavones [16]. It was originally isolated from thyme, dandelion, and sage leaves but is also present in numerous foods, such as carrots, fennel, peppers, celery, and in officinal herbs like chamomile tea [17]. The attention to this compound is due to its multiple biological properties, especially to its antioxidant and anti-inflammatory effects, as evidenced by numerous scientific studies [18,19]; in many in vitro and in vivo models, Lut has been shown to inhibit several pro-inflammatory cytokines, including tumor necrosis factor-alpha (TNF-α), and to modulate nuclear factor kappa-light-chain-enhancer of activated B cells (NF-κB) pathway, thus demonstrating the ability of flavonoids to inhibit inflammatory processes [20,21,22].

On these bases, the purpose of this work was to investigate the anti-inflammatory properties of Lut on an animal model of periodontitis induced by LPS in rats. 

## 2. Results

### 2.1. Effects of Lut Administration on Bone Destruction Induced by LPS in Gingival Tissues

**Figure 1:**
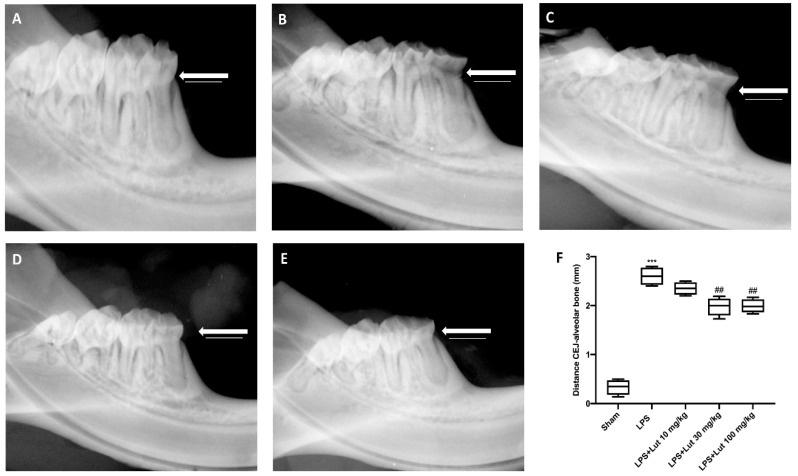
Luteolin (Lut) administration decreased the alveolar bone distance. Fourteen days after the lipopolysaccharide (LPS) injection, the X-rays of the rats LPS-induce periodontitis showed a greater distance from the cement–enamel junction (CEJ) to the bone (**B**,**F**), compared to the sham group rats (**A**,**F**). Lut 30 mg/kg (**D**,**F**) and 100 mg/kg (**E**,**F**) were effective in reducing this distance, as opposed to treatment with Lut 10 mg/kg which proved ineffective (**C**,**F**). Values reported in the box plot are expressed as mean ± SEM of 10 rats for each group. ^***^ *p* < 0.001 vs. sham; *^##^* *p* < 0.01 vs. LPS group.

In the LPS-induced periodontitis group (Figure 1B,F), the radiographic distance from the cement–enamel junction (CEJ) to the bone was considerably larger than the sham group (Figure 1A,F). Treatment with Lut at a dose of 10 mg/kg (Figure 1C,F) has proved to be ineffective for decreasing this distance, whereas the treatment with Lut at a dose of 30 (Figure 1D,F) and 100 mg/kg (Figure 1E,F) has proved to be equally effective in decreasing the alveolar bone distance.

### 2.2. Effects of Lut Administration on Histological Damage and Neutrophilic Infiltration

**Figure 2:**
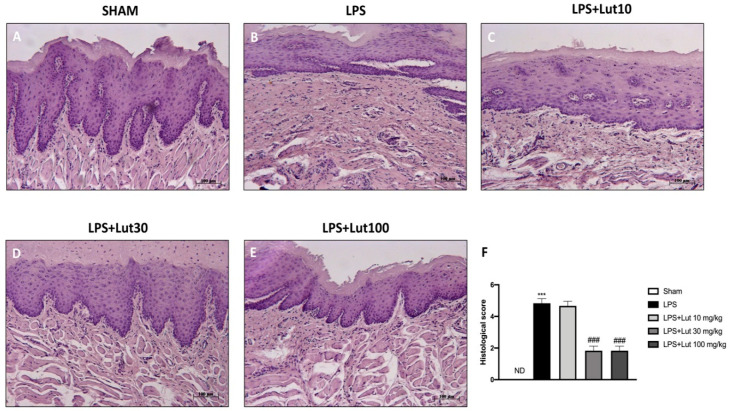
Lut administration reduced histological damage LPS-induced periodontitis. No histological damage was found in the gingivomucosal tissues from sham-group rats (**A**), see histological score (**F**). Extensive damage, accompanied by edema, tissue injury, and inflammatory cells infiltration, was assessed in LPS rats (**B**), see histological score (**F**). The administration of Lut 30 mg/kg (**D**), see histological score (**F**) and 100 mg/kg (**E**), see histological score (**F**), reduced LPS tissue damage as opposed to treatment with Lut 10 mg/kg which proved ineffective (**C**), see histological score (**F**). Data are representative of at least three independent experiments; One-Way ANOVA test. ^***^ *p* < 0.001 vs. sham; *^###^* *p* < 0.001 vs. LPS group. ND = not detectable.

Trough H/E staining the tissue integrity of each section was analyzed. No histopathological alteration was found in sham-group rats (Figure 2A, and see histological score 2F). While, histological examination of the LPS group revealed a significant increase in edema and tissue damage (Figure 2B, and see histological score 2F) that was significantly reduced after Lut 30 mg/kg and Lut 100 mg/kg administrations (Figure 2D,E, and see histological score 2F). Contrarily, rats treated with Lut 10 mg/kg still showed considerable tissue damage (Figure 2C, and see histological score 2F). 

**Figure 3:**
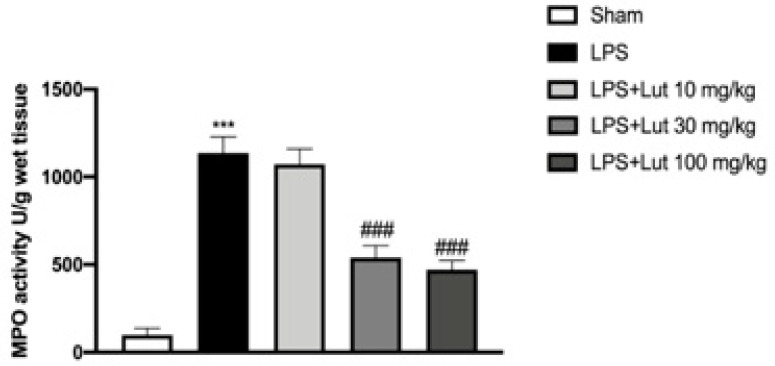
Lut treatment moderated neutrophilic infiltration. An increase in MPO levels was found in LPS-induced periodontitis rats, compared to the sham group. Only the 30 and 100 mg/kg dosages proved to be equally effective in reducing MPO levels. One-Way ANOVA test.^***^ *p* < 0.001 vs. sham; *^###^* *p* < 0.001 vs. LPS group.

Similar results were obtained from the myeloperoxidase (MPO) analysis, a marker for neutrophil infiltration. In the LPS group were revealed increased levels of MPO, whereas the two higher doses of Lut were able to markedly decrease the MPO expression; meanwhile rats treated with Lut 10 mg/kg showed MPO levels almost equivalent to the LPS group. The sham group instead revealed minimal expressions of neutrophilic infiltration (Figure 3).

Based on these results, we decided to continue our experiments with the dose of 30 mg/kg of Lut that possesses the same efficacy as the highest dose, 100 mg/kg, but with less toxicity.

### 2.3. Effects of Lut Treatment on Collagen Fibers

**Figure 4:**
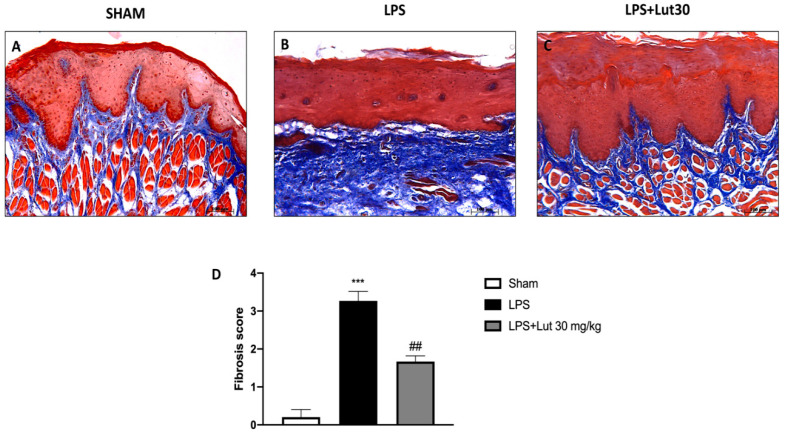
Lut treatment reduced collagen formation. Masson’s trichrome stain presented an increase in the concentration of collagen fibers in gingivomucosal tissues in the LPS group (**B**,**D**), compared to the control group (**A**,**D**). Lut 30 mg/kg significantly attenuated collagen formation (**C**,**D**). One-Way ANOVA test.^***^ *p* < 0.001 vs. sham; *^##^* *p* < 0.01 vs. LPS group.

Masson’s staining allowed us to evaluate the development of fibrous connective tissue as a repairing response to injury or damage. LPS injected rats (Figure 4B, and see fibrosis score 4D) presented an increase of collagen formation in gingivomucosal tissue sections in comparison with the sham group (Figure 4A, and see fibrosis score 4D). The increase in collagen fibers was considerably decreased by Lut 30 mg/kg treatment (Figure 4C, and see fibrosis score 4D).

### 2.4. Effects of Lut Treatment on Mast Cell Degranulation 

**Figure 5:**
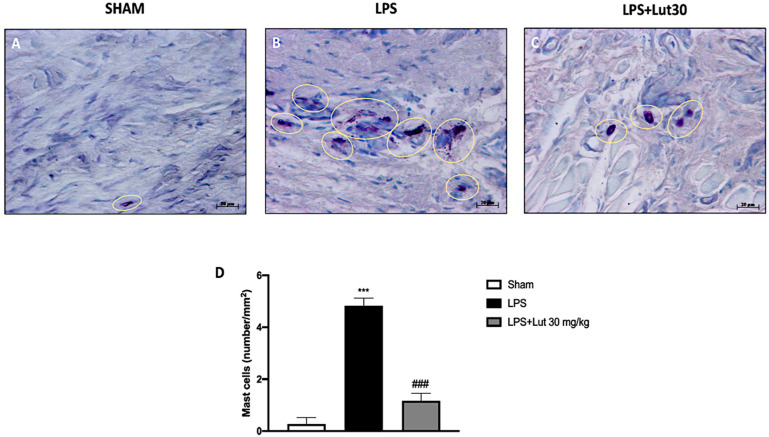
Effects of Lut treatment on mast cell degranulation. Toluidine blue staining allowed mast cell count. In gingivomucosal tissues of rats belonging to the LPS group, an increased number of mast cells was identified (**B**,**D**), as compared to control group (**A**,**D**). Lut 30 mg/kg considerably reduced mast cell infiltration (**C**,**D**). Yellow circles indicate the mast cells degranulated appeared in the tissue. One-Way ANOVA test.^***^ *p* < 0.001 vs. sham; *^###^* *p* < 0.001 vs. LPS group.

We investigated mast cell infiltration and their degranulation through toluidine blue staining. There was no full-blown inflammatory state in the gingivomucosal tissues of the sham group, as confirmed by the minimal presence of mast cells (Figure 5A,D). The group treated with LPS instead showed high levels of mast cell infiltration (as shown in Figure 5B,D); these elevated levels were extensively reduced by Lut 30 mg/kg treatment (Figure 5C,D).

### 2.5. Lut Treatment Modulated NF-κB Pathway and Pro-Inflammatory Cytokines Production 

**Figure 6:**
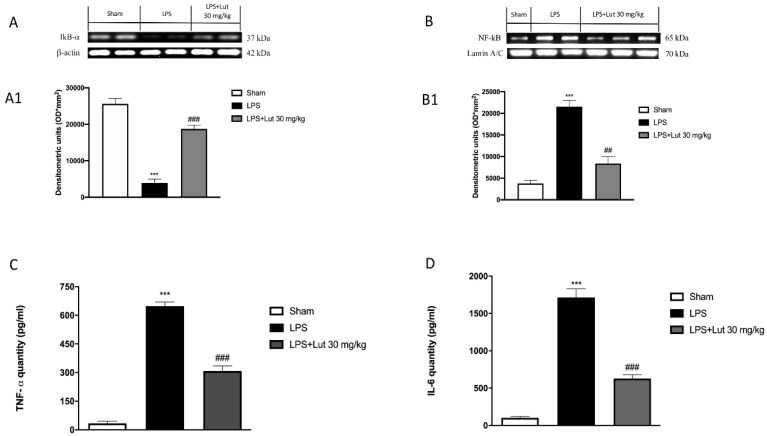
Effects of Lut treatment on NF-κB pathway and pro-inflammatory cytokines. Western blot analysis demonstrated an increase in the degradation of IκB-α in the LPS group (**A**) and densitometric analysis (**A1**) compared to the sham group (**A**) and densitometric analysis (**A1**). Lut 30 mg/kg has proven to be truly effective in restoring these levels (**A**) and densitometric analysis (**A1**). NF-κB was significantly increased in the LPS group (**B**) and densitometric analysis (**B1**), as compared to the sham group (**B**) and densitometric analysis (**B1**); Lut 30 mg/kg effectively decreased the levels of NF-κB (**B**) and densitometric analysis (**B1**). The levels of TNF-α (**C**) and IL-6 (**D**) were significantly increased in rats injected with LPS. The increases in levels of TNF-α and IL-6 were significantly attenuated in rats administrated with Lut 30 mg/kg. Data are representative of at least three independent experiments. One-Way ANOVA test.^***^ *p* < 0.001 vs. sham; *^###^* *p* < 0.001 vs. LPS group. *^##^* *p* < 0.01 vs. LPS group.

To prove the anti-inflammatory effect of Lut, we investigated, through Western blot analysis, its action on NF-κB pathway. The expression of NF-κB was found at basal levels in the sham group (Figure 6B and densitometric analysis 6B1), elevated in the LPS group (Figure 6B and densitometric analysis 6B1) and appreciably reduced by treatment with Lut 30 mg/kg (Figure 6B and densitometric analysis 6B1). In relation to this, the protein levels of IκB-α (cytosolic protein associated with NF-κB) confirmed the action of Lut in the NF-κB pathway. In fact, these levels appeared high in the sham group (Figure 6A and densitometric analysis 6A1), significantly downregulated in rats injected with LPS (Figure 6A and densitometric analysis 6A1) and remarkably restored in rats administered with Lut 30 mg/kg (Figure 6A and densitometric analysis 6A1). 

Furthermore, TNF-α, together with IL-6, plays a crucial role in establishing the inflammatory state in periodontitis; therefore, they can be considered specific markers of the disease [23]. All of these considerations led us to investigate the levels of cytokines previously mentioned. Samples from the sham group exhibited minimal levels of both cytokines (Figure 6C,D, respectively); on the other hand, such expressions were significantly increased in LPS-induced periodontitis rats (Figure 6C,D, respectively). In contrast, treatment with Lut 30 mg/kg significantly reduced TNF-α and IL-6 levels (Figure 6C,D, respectively).

### 2.6. Lut Treatment Decreased Pro-Inflammatory Enzymes Following LPS-Induced Periodontitis

The degradation of IκB-α, accompanied, consequently, by the translocation of NF-κB in the nucleus, involves the transcription of numerous proinflammatory genes, including the inducible enzymes COX-2 and iNOS, which play a fundamental role in the inflammatory response.

**Figure 7:**
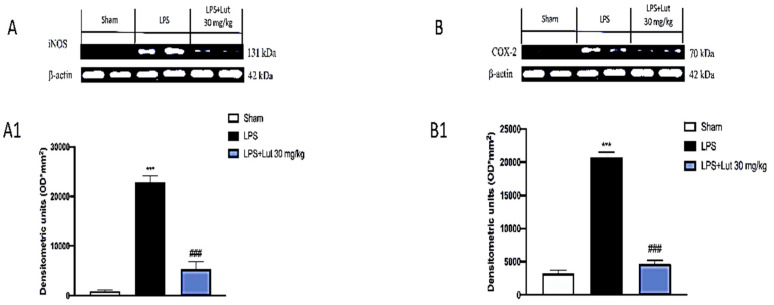
Effects of Lut treatment on pro-inflammatory enzymes. Western blot analysis of iNOS (**A**) and densitometric analysis (**A1**) and COX-2 (**B**) and densitometric analysis (**B1**) revealed minimal levels in the sham group that conversely were increased in the LPS group. Treatment with Lut 30 mg/kg proved effective to reduce COX-2 and iNOS expressions. Data are representative of at least three independent experiments. One-Way ANOVA test. ^***^ *p* < 0.001 vs. sham; *^###^* *p* < 0.001 vs. LPS group.

Lut 30 mg/kg treatment had the ability to modulate the expression of both COX-2 (Figure 7B and densitometric analysis 7B1) and iNOS (Figure 7A and densitometric analysis 7A1), compared to the damage induced by LPS (Figure 6A and densitometric analysis 6A1; Figure 7A and densitometric analysis 7A1). However, the sham-operated group shown minimal expression of both pro-inflammatory enzymes (Figure 6A and densitometric analysis 6A1; Figure 7A and densitometric analysis 7A1).

## 3. Discussion

Periodontitis is one of the most common and most serious dental diseases that causes progressive destruction of the bone surrounding the tooth; this condition, due to inflammatory processes of the marginal gingiva, is debilitating for the patient, hence the need to intervene as soon as possible through pharmacological therapy [24]. In recent years, the appreciation of natural compounds as a potential innovative treatment for human health has grown considerably [25]. 

Lut is a molecule of natural origin that is present in a large variety of fruits and vegetables and also in medicinal herbs; it has been shown to have great beneficial properties on human health [26,27,28]. Specifically, its anticancer properties are known, as shown by several studies [27,29], but it also has anti-inflammatory [20] and antioxidant effects [30]. 

Previous evidence led us to investigate the properties of this compound in an experimental model of periodontitis induced by LPS, in order to evaluate its potentiality. 

One of the hallmarks of periodontitis is alveolar bone loss; this bone destruction is due to a process, both immune and inflammatory, with which our body tries to counteract oral bacterial dysbiosis [31]. As demonstrated by our results, Lut had the ability to reduce alveolar bone loss caused by LPS injection. The most significant results were obtained exclusively at the doses of 30 and 100 mg/kg of Lut, while, at the dose of 10 mg/kg, alveolar bone loss was comparable to the LPS group. 

The pathogenic developments of inflammatory periodontal diseases are originated by subgingival plaque microflora and factors such as LPS derived from specific pathogens [31]. Locally, this inflammatory condition promotes tissue damage, thus causing the morphological alteration of the periodontium [32]. In particular, tissue damage is associated with the formation of edema and inflammatory cell infiltration with clear damage to gingivomucosal architecture [12,33]. 

Lut administration at the two highest doses (30 and 100 mg/kg) was equally able to mitigate tissue damage caused by LPS injection, as is visible from our histological analyses. 

Neutrophils constitute the primary defense system in periodontal tissues [34]; in fact, in a healthy oral cavity, populations of neutrophils tend to be para-inflammatory. On the contrary, the phenotypes of pro-inflammatory neutrophils are present in periodontal disease [35]. Lut treatment, as demonstrated by the MPO analysis, significantly reduced the presence of neutrophilic infiltration; this reduction was equally significant at the doses of 30 and 100 mg/kg, while it was ineffective, once again, at the dose of 10 mg/kg. 

Given the effectiveness of Lut 30 mg/kg in counteracting tissue damage, as already highlighted by the H&E staining, we also assessed the effect of Lut treatment on collagen fibers through Masson’s trichrome stain. In periodontitis, in fact, prolonged inflammation causes apical migration of junctional epithelium on the root surface and activates collagen destruction; specifically, degradation of type I collagen occurs in the connective tissue and periodontal ligament [33]. 

Our results clearly demonstrated that Lut 30 mg/kg was able to decrease the concentration of collagen fibers in gingivomucosal tissues. 

There is also a probable cross-talk between the increase in collagen fibers and the presence of mast cell infiltration in periodontitis [36]. Mast cells are immune cells that stimulate the inflammatory process [37] and therefore play a primary role in inflammation disease like periodontitis. Lut 30 mg/kg, as evidenced by our results, significantly reduced the degranulation of mast cells in the inflamed gingivomucosal tissues. 

The intense inflammatory condition that characterizes periodontitis includes the involvement of several pathways; in particular, the correlation between NF-κB and periodontitis is widely known, as demonstrated by several clinical studies [38,39]. Lut 30 mg/kg decreased the levels of NF-κB and increased the expression of the cytosolic protein IκB-α, as shown from Western blot analysis performed. Furthermore, it is known that the translocation of NF-κB in the nucleus promotes the transcription of pro-inflammatory genes, upregulating the expression of pro-inflammatory proteins. Western blot analysis showed that Lut administration also moderated the expression of two key enzymes of the inflammatory cascade, namely iNOS and COX-2.

Furthermore, the production of pro-inflammatory cytokines has also been related to periodontal disease. Particularly, many clinical studies [33,40,41] have demonstrated the correlation between high TNF-α and IL-6 expressions and periodontal disease, highlighting their involvement and crucial role in the evolution of gingival inflammation. As shown by our results, treatment with Lut 30 mg/kg decreased the expressions of both cytokines. 

Persistent gingivitis in young patients represents, in fact, a risk factor for periodontal attachment loss and for tooth loss in adulthood; inflammation of the gingival tissues represents not only the precursor of periodontitis but also a clinically relevant risk factor for disease progression and tooth loss [42].

Given the results obtained from this study through several methodological approaches, it is possible to affirm that Lut has good anti-inflammatory capacities in counteracting the inflammatory state caused by LPS-induced periodontitis. Therefore, Lut implementation could represent a valid natural support in the pharmacological strategy for periodontitis, thus improving the well-being of the oral cavity. Furthermore, Lut’s anti-inflammatory capabilities could open new perspectives in the field of applicability of this natural compound also in products used for the prevention of inflammatory processes of the oral cavity, like toothpaste and mouthwash; further experiments need to be carried out in more in-depth studies, to confirm this preventive applicability.

## 4. Materials and Methods

### 4.1. Materials

Unless otherwise indicated, all materials were acquired from Sigma-Aldrich Company Ltd. (St. Louis, Missouri, USA). All stock solutions were made in non-pyrogenic saline (0.9 % NaCl, Baxter, Milan, Italy). All other chemicals were of the highest commercial grade available.

### 4.2. Animals

The study was performed on Sprague-Dawley male rats (Envigo, Milan, Italy), weighing 200–230 g. They were housed in a controlled environment (22 ± 2 °C, 55 ± 15 % relative humidity, 12 h light/dark cycle), with food and water ad libitum, minimizing stress conditions.

Animal experiments complied with Italian regulations on the protection of animals used for experimental and other scientific purposes (DM 116192), as well as EU regulations (OJ of EC L 358/1, 18th December 1986). 

### 4.3. LPS-Induced Periodontitis

Periodontitis was induced as described by Reference [43] and reported below. After slightly anesthetizing the animals with sodium pentobarbital (35 mg/kg), periodontitis was induced by a single 1 µL LPS (10 μg/μL) intragingival injection derived from *Salmonella typhimurium* (Sigma-Aldrich) in sterile saline solution. The inoculation was made in the mesolateral side at the interdental papilla between the first and the second molar. It was performed slowly, and the needle was kept in place for some seconds after the injection, to guarantee that LPS was not lost through needle extraction. In addition, the animals were weighed daily, in order to control regular food intake and their masticatory behavior. 

### 4.4. Experimental Groups

Rats were randomly divided into several groups (*n* = 10 for each), as reported below:

Group 1: sham + saline: animals received a single intragingival injection of saline solution instead of LPS (*N* = 10);

Group 2: LPS + saline: rats were subjected to LPS-induced periodontitis (*N* = 10);

Group 3: LPS + Lut 10 mg/kg: rats were subjected to LPS-induced periodontitis plus daily administration of Lut (10 mg/kg) for 14 days, starting from 1 h after the injection of LPS (*N* = 10);

Group 4: LPS + Lut 30 mg/kg: rats were subjected to LPS-induced periodontitis plus daily administration of Lut (30 mg/kg) for 14 days, starting from 1 h after the injection of LPS (*N* = 10);

Group 5: LPS + Lut 100 mg/kg: rats were subjected to LPS-induced periodontitis plus daily administration of Lut (100 mg/kg) for 14 days, starting from 1 h after the injection of LPS (*N* = 10).

For oral administration, Lut was dissolved in 0.5 mL ethanol (50% purity) and given to the rats by oral gavage; the dosages of Lut were chosen on the basis of previous studies [44,45].

At the end of the experiment, 14 days after LPS injection, the animals were sacrificed, and the gums removed by surgical procedure and processed for biochemical analysis and histological examinations.

### 4.5. Radiography 

For each rat belonging to the five experimental groups, radiographic analyses were performed, using an X-ray machine (Bruker MS FX Pro, Billerica, MA, USA). The X-ray tube was operated at 30 kW, with a current of 6 mA, for 0.01 s, and the source-to-sensor distance was 50 cm. At the end of the experiment, through the radiographs, we estimated the dental alveolar bone level expressed as the distance from the cement–enamel junction (CEJ) to the maximum coronal level of the alveolar bone crest (CEJ bone distance), using IMAGE J processing software (Image J software, National Institutes of Health, Bethesda, MD, USA).

### 4.6. Histological Examination

Histological procedures were performed as previously reported by Reference [46] and described below. Samples were fixed in 10% (*w/v*) PBS-buffered formaldehyde solution at 25 °C for 24 h, after which they were dehydrated via an increasing scale of alcohols and xylene, included in paraffin, and cut under the microtome to obtain sections of 7 micrometers. After being hydrated, tissue sections were stained with Hematoxylin/Eosin (H&E, Bio-Optica, Milano, Italy). A histological injury score for gingivomucosal tissue was determined, using a semiquantitative scale that measures the subsequent morphological criteria: 0, normal gingivomucosal tissue; grade 1, minimal edema or infiltration; grade 2, moderate edema and inflammatory cell infiltration without obvious damage to gingivomucosal architecture; and grade 3, severe inflammatory cell infiltration with obvious damage to gingivomucosal architecture. For H&E staining, the results were shown at 10x magnification (100 µm scale bar). All the histological studies were performed in a blinded fashion.

### 4.7. Myeloperoxidase Activity

Myeloperoxidase (MPO) is an enzyme contained in the azurophilic granules of polymorphonuclear neutrophils and macrophages and is released in the extracellular liquid in the presence of inflammatory states. Various studies have highlighted how MPO is related to oxidative stress and inflammatory processes; its determination is, therefore, a useful biomarker for diagnostic purposes.

MPO activity was determined in gingivomucosal tissues as previously described by Reference [47]. 

Samples were homogenized in a buffer containing 0.5% hexadecyl-trimethyl-ammonium bromide dissolved in 10 mM potassium phosphate buffer, pH 7, and centrifuged for 30 min, at 20,000 rpm at 4 °C. Subsequently, the fraction “supernatant” was reacted with a solution of 1.6 vmM tetramethylbenzidine and 0.1 mM H_2_O_2_. The rate of change in absorbance was measured spectrophotometrically at 650 nm. MPO activity was measured as the quantity of enzyme degrading 1 mM of peroxide 1 min at 37 °C and was expressed in units per gram weight of wet tissue.

### 4.8. Masson Trichrome Stain 

Masson’s trichrome is a coloring particularly useful for highlighting connective tissue, collagen, reticular fibers, and muscle fibers. Thus, to assess fibrosis degree, gingivomucosal sections were stained with the Masson trichrome stain, according to the manufacturer’s instructions (Bio-Optica, Milan, Italy). For Masson trichrome staining, the results were shown at 10x magnification (100 µm scale bar). 

### 4.9. Blue Toluidine Staining

To evaluate mast cell amount and their degranulation, gingivomucosal sections were stained with toluidine blue (Bio-Optica, Milano, Italy). This basic dye colors the sections blue, highlighting the mast cells that appear purple. The number of metachromatic stained mast cells was obtained by counting five high-power fields for the section, using an Axiovision Zeiss (Milan, Italy) microscope and the correlated AxioVision software (Carl Zeiss Vision, Jena, Germany). Data were reported as the mean with standard deviation (SD). For toluidine blue staining, results were shown at 40x magnification (20 µm scale bar).

### 4.10. Western Blot Analysis for IκB-α, NF-κB, COX-2, and iNOS

Cytosolic and nuclear extracts of gingivomucosal tissues were prepared as previously described by Reference [48].

In the cytosolic fraction, the expressions of kappa light polypeptide gene enhancer in B cells inhibitor alpha (IκB-α), iNOS, and cyclooxygenase 2 (COX-2) were quantified. 

In the nuclear fraction, the expression of NF-κB was quantified. Filters were blocked with 1× PBS, 5% (*w/v*) nonfat dried milk (PM), for 40 min, at room temperature, and then probed with following antibodies: anti-IkB-α (1:500, Santa Cruz Biotechnology, Dallas, Texas, USA #sc1643), anti-NF-κB (1:500, Santa Cruz Biotechnology, #sc8008), anti-Cox2 (1:500, Santa Cruz Biotechnology, #sc-1746), anti-iNOS (1:500, Santa Cruz Biotechnology, #sc8310) in 1× PBS, and 0.1% Tween-20, 5% *w/v* nonfat dried milk (PMT) at 4 °C, overnight. After that, the membranes were incubated with peroxidase-conjugated bovine anti-mouse IgG secondary antibody or peroxidase-conjugated goat anti-rabbit IgG (1:2000, Jackson ImmunoResearch, West Grove, Pennsylvania, USA) for 1 h, at room temperature. To ascertain that blots were loaded with equal amounts of proteins, they were also incubated in the presence of the antibody against GAPDH (cytosolic fraction 1:500; Santa Cruz Biotechnology) or lamin A/C (nuclear fraction 1:500 Sigma-Aldrich Corp.), as described by Reference [49].

### 4.11. ELISA Assay for TNF- α and IL-6

ELISA assay was performed as described by Campolo M. et al. [50].

Gingivomucosal tissues were thawed on ice and homogenized in 300 μL lysis buffer (750 μL, Pierce #87787, Thermo Fisher Scientific, Waltham, MA, USA) and then complemented with a protease inhibitor cocktail (Sigma-Aldrich, Rehovot, Israel). Subsequently, the samples were homogenized and centrifuged at 14,000× *g* for 10 min at 4 °C; supernatants were collected, aliquoted, and deposited at −20 °C. Cytokines levels were measured by ELISA, according to the manufacturer’s instructions. 

### 4.12. Statistical Analysis

All values are showed as mean ± standard error of the mean (SEM) of N observations. N denotes the number of animals employed. The experiment is representative of at least three experiments performed on different days on tissue sections collected from all animals in each group. Data were analyzed by one-way ANOVA, followed by a Bonferroni post hoc test for multiple comparisons. A *P*-value of less than 0.05 was considered significant.
